# Subtype-specific functions of basal IFNλs

**DOI:** 10.1128/jvi.01755-25

**Published:** 2026-06-02

**Authors:** Megan L. Stanifer

**Affiliations:** 1 Department of Molecular Genetics and Microbiology, University of Florida College of Medicine 12233, Gainesville, Florida, USA; New York University Department of Microbiology, New York, New York, USA

**Keywords:** interferon, type III interferon, IFNλ, epithelial cells, basal gene expression

## Abstract

Type III interferons (IFNλs) represent the newest interferon family, comprising four human subtypes (IFNλ1–4) that signal through the tissue-restricted IFNLR1 receptor. While traditionally characterized as antiviral cytokines protecting mucosal barriers, accumulating evidence suggests that IFNλs possess broader physiological roles that remain incompletely understood. A critical gap in our knowledge concerns whether the four IFNλ subtypes function redundantly, or serve distinct biological purposes. Recent discoveries challenge the assumption of functional redundancy among IFNλ subtypes. Emerging data indicate that different subtypes exhibit distinct signaling kinetics, potencies, and downstream effects on epithelial biology. Beyond their established antiviral functions, IFNλs appear to regulate fundamental aspects of epithelial homeostasis, including barrier integrity, cellular differentiation, and tissue architecture. The discovery of constitutive basal IFNλ expression in healthy epithelia—driven by microbiota and endogenous danger signals—suggests that these cytokines continuously shape tissue physiology rather than functioning solely as inducible defense molecules. Understanding subtype-specific IFNλ functions has become increasingly urgent as these cytokines enter clinical development. The tissue-restricted expression of IFNLR1 offers therapeutic advantages over broadly-acting type I interferons, but optimal clinical application requires comprehensive knowledge of how individual subtypes influence both pathogen control and tissue homeostasis. Critical questions remain: Do specific subtypes preferentially regulate barrier function versus antiviral immunity? Can imbalanced subtype expression contribute to epithelial pathology? How do pathogens differentially induce IFNλ subtypes to evade immunity or promote tissue damage? Addressing these questions will illuminate fundamental principles of mucosal immunity and guide rational design of IFNλ-based therapeutics that maximize protection while preserving epithelial health.

## INTRODUCTION

The interferon (IFN) system represents a fundamental component of vertebrate antiviral defense mechanisms. These cytokines, originally identified in the 1950s due to their capacity to inhibit viral propagation, constitute three major families (type I, type II, and type III interferons) and are distinguished by their molecular characteristics, receptor specificity, and functional properties (1, 2). While all interferon classes contribute to pathogen resistance, each exhibits specialized roles in immune regulation and antiviral protection (3). Research employing comparative genomics has revealed that different interferon families—and even individual members within each family—have experienced unique selective pressures throughout evolution (4, 5). This differential evolutionary trajectory indicates that these molecules serve both overlapping and specialized functions in host defense mechanisms (5). This review will focus on the newest member of the interferon family, the type III interferons, their recently identified non-canonical roles in regulating epithelial barrier function, and how each type III interferon acts in a unique subtype-specific manner (6, 7).

## DISCOVERY AND CLASSIFICATION OF TYPE III INTERFERONS

**Fig 1:**
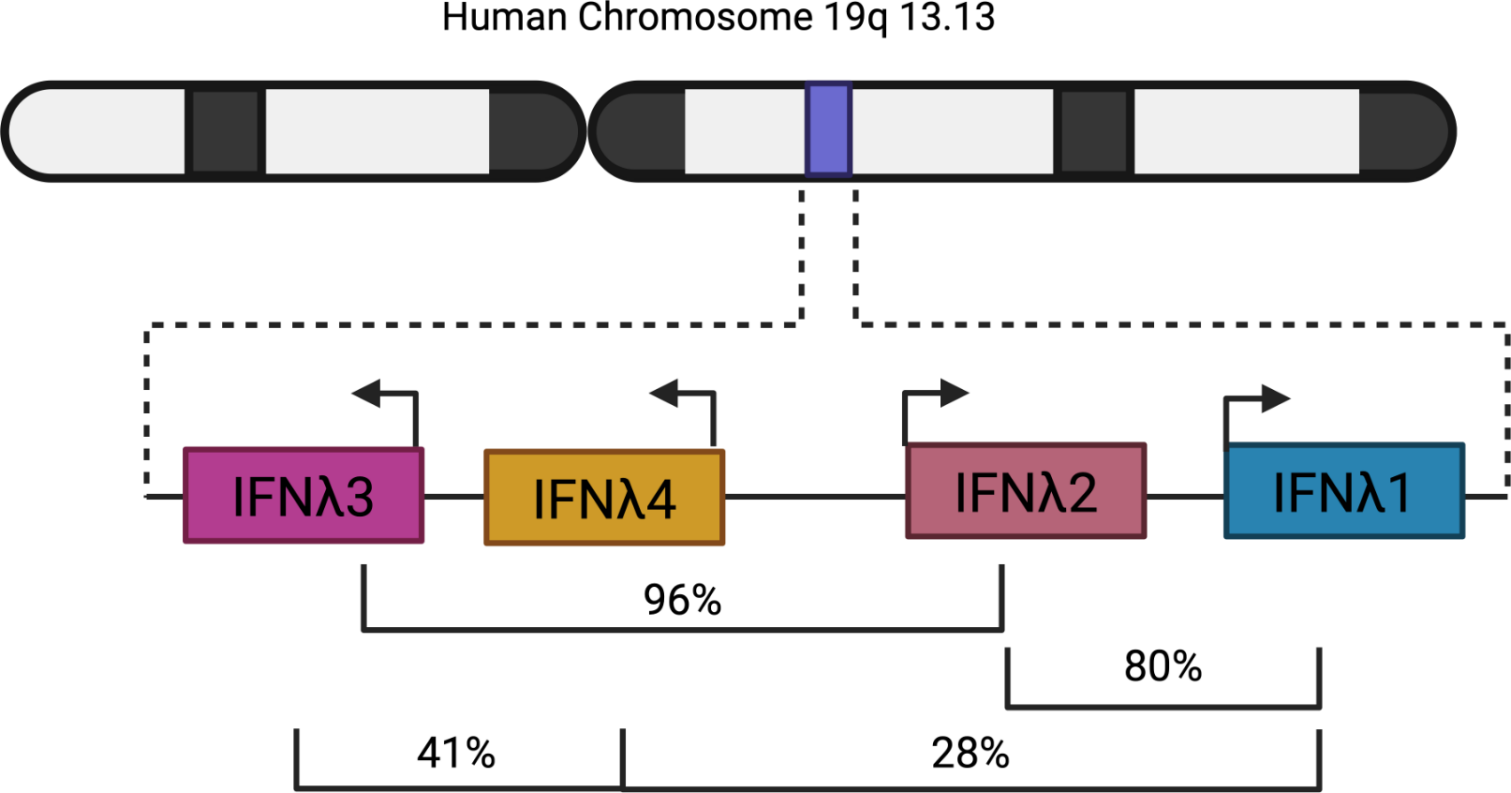
Diagram of human chromosome 19 and the IFNλ genes locus. There are four IFNλs, and their sequence similarity is denoted under each gene. Created in BioRender (https://BioRender.com/2tqolez).

Type III interferons, also known as IFN-lambdas (IFNλs), represent the newest addition to the interferon superfamily, having been characterized in 2003 (8, 9). Human type III interferons include four distinct proteins: IFNλ1 (alternatively termed IL-29), IFNλ2 (IL-28A), IFNλ3 (IL-28B), and IFNλ4. These genes cluster within a confined region on chromosome 19 (10) (Fig. 1). Initial identification of IFNλ1–3 resulted from bioinformatic analyses targeting IL-10-like sequences (8, 9). However, amino acid sequence comparisons reveal limited similarity between IFNλs and either IL-10 (11–13% identity) or type I interferons (15–19% identity) (9). The fourth family member, IFNλ4, was discovered 10 years later, and its expression depends on a specific genetic polymorphism (rs368234815) that maintains an intact coding sequence (11). Notably, numerous individuals possess a variant containing a frameshift mutation that abolishes IFNλ4 production, resulting in substantial population heterogeneity (12). The expression of IFNλ4 is often linked to genetic ancestry, where 95% of Africans express IFNλ4, while only 50% of Europeans and 15% of Asians express IFNλ4 (13). Intriguingly, individuals producing functional IFNλ4 demonstrate impaired hepatitis C virus elimination and diminished therapeutic responses to interferon treatment (14).

Among type III interferons, IFNλ2 and IFNλ3 display remarkable conservation, maintaining approximately 96% sequence similarity throughout mammalian evolution (9, 15, 16). These proteins exhibit broad species distribution, contrasting sharply with IFNλ1. While several species express IFNλ1, their sequences are quite divergent (17, 18). The IFNλ1 found in humans is only closely related to IFNλ1 found in primates (16). Rodents possess pseudogenes for both IFNλ1 and IFNλ4 that lack functionality (16). Despite retaining roughly 81% homology with IFNλ2/3, the restricted expression of IFNλ1 implies that it contains specialized adaptive functions (9, 15). IFNλ4 shows a closer phylogenetic relationship to IFNλ3 yet shares merely 41% sequence homology (11). Despite these differences in sequence homology, much work has considered IFNλ1, IFNλ2, and IFNλ3 to be functionally redundant. This is surprising as the difference in type I interferon subtypes has been well documented. Interferon α (IFNα) subtypes exhibit distinct expression patterns based on transcription factor activation—conserved subtypes (like IFNα1, -α2, -α8) are induced early, while variant subtypes (IFNα4, -α5, -α6, -α7, -α10, -α14, -α16, -α17, and -α21) are expressed later through positive feedback amplification (19–21). Functionally, these subtypes differ in their receptor-binding affinities and signaling potency, with some studies suggesting that specific subtypes may be preferentially induced by particular pathogens and elicit unique antiviral or immunomodulatory responses (19–21). This differential expression and function allow the type I interferon response to be fine-tuned based on the nature and intensity of the viral threat (19–21). This suggests that IFNλ subtypes may also exhibit unique expressions and functions.

### Interferon expression and signaling

Like their type I IFN counterparts, IFNλs activate antiviral transcriptional programs that limit pathogen spread (1, 2, 22–30). Following pathogen entry into cells, their nucleic acids or protein components are detected by intracellular pattern recognition receptors (PRRs), such as Toll-like receptors (TLRs), RIG-I-like receptors (RLRs), or cyclic GMP-AMP synthase (cGAS) (31–33). Pathogens can also be sensed from the outside of the cell by surface-expressed TLRs. Upon sensing pathogens, TLRs, RLRs, and cGAS elicit a signaling cascade that results in the phosphorylation of interferon regulatory factors (IRF) 3 and 7 (31–33). Phosphorylated IRF3/7 dimerize and translocate to the nucleus where they drive the expression of IFNλ1–3 (31–33). Following transcription and translation, IFNλs are secreted from the cell where they can act in an autocrine or paracrine manner (1, 2, 30). IFNλs induce the expression of downstream genes through the binding of a heterodimeric receptor complex consisting of IFNLR1 and IL-10Rb (1, 2, 30, 34). While IL-10Rb is ubiquitously expressed, IFNLR1 has a more restricted expression and is found mainly on epithelial cells and a limited number of immune cells (35, 36). As such, IFNλs function as essential mediators of innate immunity, particularly at mucosal barriers where numerous pathogens initially encounter host tissues (1, 2, 30, 35). Following binding of IFNλs to IFNLR1/IL-10Rb, a second signaling cascade is induced, which results in the phosphorylation of STAT1 and STAT2 (2, 34). Phosphorylated STAT1/2 bind to IRF9, forming the ISGF3 complex, which translocates into the nucleus and drives the expression of hundreds of interferon-stimulated genes (ISGs) (2, 34).

### Antiviral properties of interferons

Much of the preliminary characterization of IFNλs has focused on their antiviral properties. IFNλs were found to activate similar ISGs as type I interferons, which led to the initial speculation that they were redundant (1, 2, 24, 25, 30). However, it has since been found that each interferon family plays a specific role in controlling virus spread and pathogenesis in murine models, often due to the unique distribution of their receptors (1, 2, 24, 25, 30). In addition, *in vitro* experiments have shown that while both type I interferons and IFNλs induce ISGs, they differ in the magnitude and kinetics of induction (25, 37–39). Most of the ISGs induced by type I and type III IFNs are overlapping; however, type I interferons induce more ISGs faster and to a higher magnitude than IFNλs, which usually take longer to reach a peak of ISG induction (25). This leads to unique antiviral environments, as many of the ISGs are expressed in different combinations. Importantly, IFNλs have been found to be maintained longer in cell culture and provide extended protection against virus infections (23). In addition, IFNλs have been found to be the key cytokine to protect epithelial surfaces. Mice lacking the IFNLR1 receptor are more susceptible to enteric virus infections (e.g., rotavirus [27, 40–42], reovirus [43], and norovirus [27–29]), upper airway infections (e.g., influenza [44, 45]), congenital infections (e.g., ZIKA [46, 47]), and have increased blood-brain barrier permeability (48) than wild-type mice or mice lacking the type I interferon receptors (IFNAR1/2). Additionally, using similar receptor depletion techniques, cell culture and organoid models have also shown that IFNλs are key to protecting human intestinal, hepatic, and respiratory cells against a wide range of viruses, such as reovirus, rotavirus, and SARS-CoV-2 (22–24, 26, 49). Together, this work has shown that IFNλs have distinct functions from type I interferons. However, whether all IFNλs act in a similar or distinct manner to control virus infection has only started to be addressed.

Previous work from our lab has started to evaluate subtype-specific differences in IFNλs’ antiviral properties. We examined the antiviral capacity of IFNλ3 and IFNλ4 in both intestinal and hepatic cells (50). We found that IFNλ4 exhibits distinct signaling kinetics compared to IFNλ3, inducing a more rapid yet transient antiviral response characterized by faster STAT1 phosphorylation and ISG induction, contrasting with the slower, sustained signaling of IFNλ3 (50). In addition, we evaluated naturally occurring human variants and found that these variants modified the magnitude of IFNλ4 activity. The common P70S variant reduced IFNλ4 potency, while the rare ancestral K154E variant enhanced IFNλ4 to IFNλ3-like levels (50). Importantly, IFNλ4’s rapid, transient signaling profile more closely resembles type I IFNs despite utilizing the same IFNλR1/IL10R2 receptor complex as other IFNλs, and these kinetic differences are conserved across non-human primates (50). Together with genetic associations linking IFNλ4 variants to infectious disease outcomes, these findings support the hypothesis that IFNλ4 has evolved non-redundant, specialized immunological functions distinct from IFNλ1–3.

### Anti-pathogen properties of interferons

Beyond its well-established antiviral functions, IFNλ has emerged as a broader regulator of mucosal immunity with increasingly recognized roles in antibacterial and antifungal defense that have been reviewed in detail elsewhere (51). Briefly, following bacterial infections, IFNλs appear to be preferentially induced by microbe-associated molecular patterns (MAMPs) via TLR2, TLR5, and MyD88-dependent TLR4 signaling. This drives the expression of ISGs with direct antibacterial effector functions, including IFITM proteins, viperin, and guanylate-binding proteins (52). In addition, at the epithelial barrier, IFNλ1 was found to reinforce tight junction integrity and prevent transmigration of invasive enteric pathogens, such as *S. flexneri* and *Salmonella* Typhimurium (53). Uniquely among IFNλ family members, IFNλ4 has been shown to function as a constitutively expressed intestinal antimicrobial protein capable of directly killing bacteria through LPS binding, self-assembly into bacteria-surrounding nanoparticles, and membrane disruption in porcine models (54). Interestingly, these activities were found to be entirely independent of canonical cytokine-receptor signaling (54). IFNλ also coordinates antifungal immunity, acting directly on neutrophils to drive their antifungal effector functions during invasive aspergillosis, with neutrophil-specific loss of IFNLR1 rendering mice fatally susceptible to infection (55). Together, these findings position IFNλ as a pleiotropic mucosal immune effector whose reach extends well beyond antiviral defense, although the full scope of its non-antiviral functions, whether all subtypes play a role in controlling bacterial and fungal infections, and the mechanisms underlying them remain important areas for future investigation.

## BASAL INTERFERON SIGNALING

### Expression of basal interferons

While interferons traditionally function as pathogen-induced cytokines, recently it has been found that they are also expressed continuously at low levels in uninfected tissues. This constitutive interferon activity, termed basal or tonic interferon expression, creates an environment that shapes cellular physiology, maintains tissue homeostasis, and primes immune defenses (56–58). Understanding basal interferon biology has revealed fundamental roles beyond classical antiviral immunity. Despite its minimal amplitude, this baseline signaling profoundly influences immune competence and cellular functions (59, 60). The discovery of basal interferon signaling challenged the traditional view of interferons as purely inducible defense molecules (58). Instead, cells exist along a spectrum of interferon activation, with basal signaling representing the ground state from which rapid responses can be launched (58). This paradigm shift has important implications for understanding tissue physiology, chronic inflammatory diseases, and cancer biology (56–58). Currently, most work has evaluated the expression and consequences of basal type I interferons, and understanding basal expression of IFNλs is in its infancy.

The maintenance of basal interferon primarily results from two key sources of stimulation: commensal microbiota and endogenous damage-associated molecular patterns (DAMPs). At mucosal surfaces, particularly in the intestine, the vast community of resident microorganisms provides constant low-level stimulation of pattern recognition receptors (28, 29, 42, 58). Bacterial cell wall components, nucleic acids, and metabolites engage Toll-like receptors and cytosolic sensors, triggering modest but persistent interferon production (58). This microbiota-driven signaling explains why germ-free mice exhibit dramatically reduced basal interferon levels and increased susceptibility to various pathogens (28, 42). The relationship appears carefully calibrated—sufficient microbial sensing maintains protective interferon levels without triggering harmful inflammation.

Endogenous DAMPs represent the second major driver of basal interferon signaling. During normal cellular processes such as proliferation, differentiation, and turnover, cells release various self-derived molecules that can activate interferon pathways (56, 57, 61). Mitochondrial DNA released during normal metabolic stress, extracellular ATP from dying cells, and self-nucleic acids from cellular debris all contribute to baseline interferon production (56, 57, 61). The cGAS-STING pathway particularly responds to cytosolic self-DNA, maintaining interferon expression even in sterile conditions (56, 57, 61). This DAMP-driven signaling becomes especially important in metabolically active tissues and during physiological stress, creating a feedback system where cellular state influences immune readiness through basal interferon modulation (56, 57, 61).

### Basal type I IFNs

In steady state, various cell types produce low levels of type I IFNs, maintaining both basal ISG expression and tissue homeostasis. Basal type I interferon signaling maintains low-level constitutive expression of STATs and ISGs, priming cells for rapid antiviral responses without causing overt activation. This basal interferon expression and signaling establish a baseline pool of STAT proteins and keep core ISG transcripts at detectable but sub-inflammatory levels, providing a state of immune readiness. Upon infection, this pre-existing reservoir of signaling components enables faster and more robust ISGF3 formation and ISG induction compared to naive cells, effectively lowering the threshold for mounting an effective antiviral response. Hematopoietic cells, particularly plasmacytoid dendritic cells, contribute significantly to systemic basal type I interferon levels (62–64). This continuous low-level signaling regulates hematopoietic stem cell quiescence, preventing excessive proliferation while maintaining regenerative capacity (62–64). In the bone marrow niche, basal IFNα signaling influences megakaryocyte maturation and platelet production, demonstrating roles beyond pathogen defense (65, 66). The nervous system also exhibits basal type I interferon activity that regulates synaptic plasticity and neurodevelopment (67). Microglia and astrocytes produce low levels of IFNβ that influence neuronal gene expression and circuit formation (67, 68). Dysregulation of this basal signaling contributes to neuroinflammatory conditions and may impact cognitive function (69).

Basal type I IFN signaling represents a double-edged mechanism. While providing essential antiviral protection and tissue homeostasis, dysregulated constitutive signaling contributes to autoimmunity and chronic inflammation (70, 71). Basal type I interferon levels result from complex regulatory networks involving transcription factors, epigenetic modifications, and negative feedback mechanisms. The transcription factor IRF1 particularly contributes to constitutive interferon expression (37, 72, 73), while proteins such as USP18 prevent excessive basal type I interferon signaling (74–76). Dysregulated basal interferon signaling contributes to various pathologies. Type I interferons (IFNα/β) drive chronic disorders and autoimmunity when dysregulated, with systemic lupus erythematosus (SLE) serving as the classic example, in which chronic IFNα production creates self-perpetuating cycles of B cell activation, autoantibody production, and immune complex formation that further worsen the disease (77). Monogenic type I interferonopathies, such as Aicardi-Goutières syndrome (70, 71) and STING-associated vasculopathy (78), demonstrate how constitutive IFN production from mutations in nucleic acid sensing pathways causes devastating neurological damage and systemic inflammation. Similarly, Sjögren’s syndrome (79), dermatomyositis (80, 81), and psoriasis (82, 83) each display prominent type I IFN signatures that correlate with disease severity and contribute to their distinct pathologies. Understanding these pathways offers therapeutic opportunities for enhancing antiviral immunity or dampening pathological inflammation.

### Basal IFNλs

Recently, we and others discovered that, similar to type I IFNs, IFNλs are also expressed at basal levels in epithelial cells and immune cells (6, 7, 42, 84, 85). This basal IFNλ can also be induced by either microbiota-driven mechanisms or through the sensing of DAMPs. Microbiota-driven IFNλ production has been shown to generate highly localized pockets of ISG expression in the intestinal epithelium that provide strategic, preemptive antiviral protection (42, 84, 85). These discrete pockets are restricted to individual villi or small groups of neighboring villi and arise through TLR-mediated sensing of bacterial products by hematopoietic immune cells, which then secrete IFNλ to stimulate surrounding epithelial cells (42, 84, 85). The spatial distribution of these homeostatic pockets correlates with local bacterial abundance in the intestinal lumen (42, 84, 85). Critically, this localized homeostatic IFNλ response is both necessary and sufficient for controlling enteric viral infections such as murine rotavirus (85). The pockets are rapidly ablated by antibiotic treatment or IFNλ receptor knockout, demonstrating their dependence on microbiota-derived signals (42). This spatially restricted immune surveillance mechanism allows the intestine to maintain preemptive antiviral defenses at sites of potential pathogen entry while avoiding the metabolic costs and potential tissue damage of widespread immune activation across the entire epithelium.

In addition, work from our lab revealed that basal IFNλ expression in human epithelial cells is regulated by DAMP signaling. We found that cell confluency drives the induction of basal IFNλ expression through an intricate interplay between the cGAS-STING and Hippo signaling pathways (6). As epithelial cells become confluent and polarize, they exhibit enhanced phosphorylation of TBK1, IRF3, and STAT1, leading to the increased expression of IFNλ2/3 and ISGs (6). Importantly, we demonstrated that this density-dependent immune induction is specifically mediated by IFNλs rather than type I interferons, as IFNAR knockout cells continue to express density-dependent ISGs, while IFNLR knockout cells lose ISG expression (6). Mechanistically, we uncovered that the cGAS-STING pathway senses cytosolic mitochondrial DNA to drive IFNλ expression at high cell density (6). Intriguingly, while mtDNA levels in the cytosol remain constant regardless of cell density, the Hippo pathway acts as a molecular switch—at low density, active YAP/TAZ proteins inhibit TBK1 phosphorylation and suppress the immune response, while at high density, YAP/TAZ phosphorylation relieves this inhibition (6).

**Fig 2:**
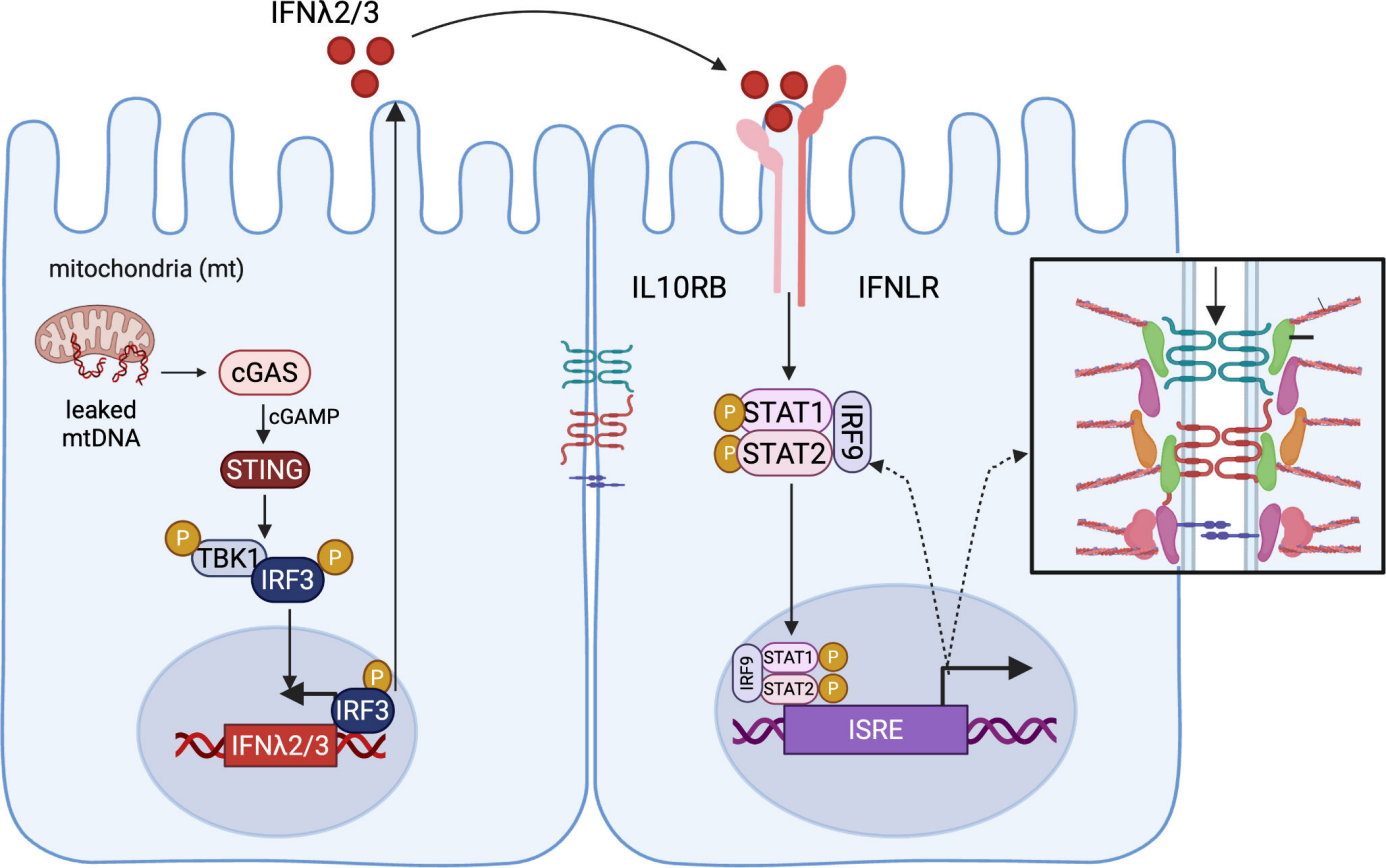
Schematic showing that IFNλ2/3 are induced through the sensing of leaked mitochondria DNA (mtDNA) by the cGAS/STING pathway in human intestinal epithelial cells. Upon expression of IFNλ2/3, they are secreted and signal to neighboring cells to induce hundreds of interferon-stimulated genes (ISGs). These basal ISGs are key for both keeping cells in an immune-ready state by ensuring sufficient amounts of important effectors, such as STAT1, STAT2, and STING. In addition, this basal IFNλ2/3 is key to regulate barrier function by downregulating claudin-2 expression, which promotes a tight epithelial barrier integrity. Created in BioRender (https://BioRender.com/4kzyki2).

Interestingly, we found that the functional significance of basal IFNλ signaling extends beyond antiviral immunity to regulate epithelial barrier integrity. We found that cells lacking IFNλ2/3 or IFNLR exhibit impaired tight junction formation and increased barrier permeability to FITC-dextran (6). Transcriptomic analysis identified claudin-2 as a key downstream target of IFNλ signaling (6). Claudin-2 is often thought of as a “leaky” tight junction protein, which is known to be involved in the formation of pore complexes. We found that claudin-2 is normally downregulated as cells polarize but remained elevated in IFNλ2/3-deficient cells. Remarkably, silencing claudin-2 in IFNλ2/3 knockout cells restores barrier function, establishing a novel regulatory axis, where STING-mediated IFNλ2/3 signaling maintains epithelial barrier integrity by suppressing claudin-2 expression (Fig. 2) (6). These findings begin to reveal the nuanced mechanisms by which epithelial cells integrate cell density cues to coordinate both immune readiness and barrier function. In addition, this work begins to shed light on how basal IFNλ expression can play key non-viral roles at epithelial surfaces.

In support of the key role that IFNλ2/3 play on barrier integrity, recent work has identified IFNλ variants as potential monogenic contributors to very early onset inflammatory bowel disease (VEOIBD) (86). Two unrelated pediatric patients presenting with bloody diarrhea in early infancy were found to harbor rare homozygous or compound heterozygous mutations in the IFNλ2 and IFNλ3 genes (86). Functional characterization revealed that these variants impair barrier function, IFNλ signaling through defective binding to IFNLR1, and reduced antiviral capacities (86). These findings implicate defects in IFNλ-mediated antiviral immunity in the pathogenesis of intestinal inflammation, representing a novel mechanism distinct from the previously identified monogenic causes of VEOIBD.

## SUBTYPE-SPECIFIC DIFFERENCES IN IFNλS

The IFNλ genes are regulated by distinct promoter architectures that underlie their differential expression patterns. The *IFNL1* promoter requires both proximal IRF3/7-binding elements near the transcription start site and a distal cluster of NF-κB-binding sites for maximal induction (87). It was found that these critical NF-κB sites are derived from transposable elements of the Alu and LTR families, revealing that *IFNL1* regulatory evolution has been shaped by genomic repeat elements in a way not shared by *IFNL2* or *IFNL3* (87). In contrast, the *IFNL2* and *IFNL3* promoters are highly similar to each other and are largely co-regulated, driven primarily by IRF3 and IRF7 without the same dependence on distally embedded NF-κB elements (87, 88). The *IFNL4* promoter is the most divergent of all (89). Although it is highly conserved across mammals and functional, it critically lacks a canonical IRF3-binding site, which causes it to only be minimally induced by virus infection (12). Expression of *IFNL4* is further gated at the level of the open reading frame by the rs368234815 dinucleotide polymorphism (ΔG/TT), restricting IFNλ4 protein production to individuals carrying the ΔG allele (11). Together, these findings highlight the remarkable regulatory diversity within the IFNλ gene family, and future studies systematically comparing *IFNL1*, *IFNL2/3*, and *IFNL4* promoter activity across relevant cell types will be essential for understanding how each family member contributes to tissue-specific antiviral and antimicrobial defense.

While genetically distinct, IFNλ1–3 have often been considered as being redundant. Most labs have not performed a systematic comparison of the activities of IFNλ1–4 and have often only evaluated the induction or antiviral properties of a single subtype. However, recent work from our lab has found that each IFNλ subtype exhibits distinct antiviral and homeostatic properties (6, 7). While IFNλs are key to priming cells against future pathogen attacks, there appears to be a functional hierarchy among IFNλ subtypes in human intestinal epithelial cells. Our work demonstrates that IFNλ2/3, but not IFNλ1, are the primary mediators of antiviral protection. Genetic deletion experiments revealed striking differences in the endogenous functions of IFNλ1 vs IFNλ2/3; cells lacking IFNλ2/3 exhibited dramatically enhanced susceptibility to diverse viruses (VSV, rotavirus, reovirus, and vaccinia virus), whereas IFNλ1 knockout cells maintained viral resistance comparable to wild-type cells. This differential susceptibility stems from the distinct roles these subtypes play in maintaining basal interferon signaling—IFNλ2/3 sustain constitutive expression of key ISGs and signaling components such as STAT1, IRF7, and RIG-I through STING-dependent mechanisms, while IFNλ1 contributes minimally to this basal antiviral state. The dominance of IFNλ2/3 in epithelial immunity was further confirmed through complementary approaches including conditioned media transfer experiments, antibody neutralization studies, and cytokine reconstitution assays. Notably, neutralization of either IFNλ2 or IFNλ3 individually resulted in only partial rescue of basal ISG expression and antiviral protection. This indicates that despite IFNλ3’s higher receptor-binding affinity relative to IFNλ1, the presence of IFNλ3 alone, when IFNλ2 has been depleted, is insufficient to fully sustain antiviral capacity. Together, these findings suggest that IFNλ2 and IFNλ3 are functionally redundant but with incomplete compensation, and that receptor-binding affinity alone does not account for all downstream effector functions. These findings establish that IFNλ2 and IFNλ3 function as the principal guardians of epithelial antiviral immunity, maintaining cellular readiness against viral challenges through constitutive signaling, while IFNλ1 plays only a minor supportive role despite being induced during infection. Combined with data showing that IFNλ4 signaling and antiviral gene induction more closely mirrors type I interferons than IFNλs (50), this work underscores the importance of dissecting the individual roles of each IFNλ subtype.

## FUTURE PERSPECTIVES

While basal type I interferons have been extensively characterized for their role in cellular homeostasis and immune surveillance, our understanding of basal IFNλ functions remains limited. Emerging evidence suggests that constitutive IFNλ expression prepares epithelial cells for pathogen encounters, maintaining a state of antiviral readiness at barrier surfaces. However, the functions of basal IFNλs likely extend beyond antiviral defense. Studies in the intestine have demonstrated that IFNλ signaling is essential for epithelial barrier integrity and tissue homeostasis. Whether other epithelial tissues similarly depend on basal IFNλ activity for fundamental cellular processes remains an open and critical question that warrants investigation. In addition, as excessive type I IFNs have been shown to be pathogenic, whether some or all IFNλs at high or continuous levels can also lead to pathology has not yet been explored.

The functional complexity of IFNλ is further underscored by the existence of four IFNλ subtypes in humans, which appear to have divergent biological roles. Our recent work has revealed that IFNλ1 functions differently from IFNλ2 and IFNλ3, where IFNλ2 and IFNλ3 appear particularly important for establishing antiviral readiness and barrier functions in epithelial cells while IFNλ1 seems to be dispensable. Given that IFNλ2 and IFNλ3 display functional redundancy yet cannot fully compensate for one another, it is likely that each subtype carries out distinct functions that have yet to be characterized. Further work dissecting the mechanisms governing their individual expression and the unique features of their downstream signaling will be essential to uncovering these subtype-specific roles. Maintaining the appropriate balance of IFNλ subtype expression and signaling appears crucial for epithelial health, as disruption of this equilibrium may contribute to tissue damage. Importantly, different pathogens may induce distinct patterns of IFNλ subtype expression, potentially driving imbalances that compromise barrier function and promote pathology. Understanding how cells regulate this balance, and how pathogens perturb it, represents a critical open topic in mucosal immunity research.

While IFNλs are broadly recognized for their protective roles at epithelial barriers, accumulating evidence demonstrates that IFNλ signaling can also disrupt epithelial homeostasis and impair tissue repair under certain conditions. Recently, it was demonstrated that IFNλ produced during respiratory infection delays lung epithelial regeneration, revealing a fundamental trade-off between antiviral defense and tissue recovery (90, 91). In addition, this delayed repair led to increased risk of bacterial superinfection following virus-induced damage (90, 91). Beyond the lung, IFNλ signaling has also been shown to impair mucosal repair in the gastrointestinal tract through the induction of pyroptosis in gut epithelial cells (92). This is particularly relevant in the gastrointestinal tract, given the rapid turnover demands of the intestinal epithelium and the potential consequences of epithelial cell death for barrier integrity. Together, these studies highlight that the timing, magnitude, and cellular context of IFNλ responses critically determine whether the outcome is protective or pathological and that sustained or dysregulated IFNλ signaling may actively contribute to tissue damage rather than resolve it.

The clinical development of IFNλ-based therapeutics underscores the urgent need to fully elucidate the distinct properties of each subtype. IFNλs have begun to enter clinical use for antiviral therapy, demonstrating promise due to their tissue-restricted activity and reduced systemic side effects compared to type I interferons (93–95). However, given the non-redundant and sometimes opposing functions of IFNλ subtypes, comprehensive characterization of their unique properties is essential to optimize therapeutic applications. Future clinical strategies must consider not only which IFNλ subtype to administer, but also appropriate dosing regimens and potential combinations that maximize antiviral protection while minimizing off-target effects and tissue damage. Without a deeper understanding of how individual IFNλ subtypes influence both pathogen control and epithelial homeostasis, we risk therapeutic approaches that inadvertently disrupt the delicate balance required for barrier integrity and tissue health.
